# Nesfatin-3 possesses divalent metal ion binding properties, which remain hidden in the nucleobindin-2 precursor protein

**DOI:** 10.1186/s12964-023-01181-6

**Published:** 2023-06-29

**Authors:** Anna Skorupska-Stasiak, Dominika Bystranowska, Józef Ba Tran, Artur Krężel, Andrzej Ożyhar

**Affiliations:** 1 Department of Biochemistry, Molecular Biology and Biotechnology, Faculty of Chemistry, Wrocław University of Science and Technology, Wybrzeże Wyspiańskiego 27, 50 370 Wrocław, Poland; 2 Department of Chemical Biology, Faculty of Biotechnology, University of Wrocław, Joliot-Curie 14a, 50-383 Wrocław, Poland

**Keywords:** Mg^2+^/Ca^2+^Zn^2+^-binding proteins, Metal ion-induced conformational change, Nesfatins, Nucb2, Proteolytic processing of the precursor proteins

## Abstract

**Background:**

Nucleobindin-2 (Nucb2) is a multidomain protein that, due to its structure, participates in many physiological processes. It was originally identified in several regions of the hypothalamus. However, more recent studies have redefined and extended the function of Nucb2 far beyond its initially observed role as a negative modulator of food intake.

**Results:**

Previously, we described Nucb2 as structurally divided into two parts: the Zn^2+^-sensitive N-terminal half and the Ca^2+^-sensitive C-terminal half. Here, we investigated the structural and biochemical properties of its C-terminal half, which, after posttranslational processing, yields the formation of a fully uncharacterized peptide product known as nesfatin-3. Nesfatin-3 likely contains all the key respective structural regions of Nucb2. Hence, we expected that its molecular properties and affinity toward divalent metal ions might resemble those of Nucb2. Surprisingly, the obtained results showed that the molecular properties of nesftain-3 were completely different from those of its precursor protein. Moreover, we designed our work as a comparative analysis of two nesfatin-3 homologs. We noticed that in their apo forms, both proteins had similar shapes and existed in solution as extended molecules. They both interacted with divalent metal ions, and this interaction manifested itself in a compaction of the protein molecules. Despite their similarities, the differences between the homologous nesfatin-3s were even more informative. Each of them favored interaction with a different metal cation and displayed unique binding affinities compared either to each other or to Nucb2.

**Conclusions:**

The observed alterations suggested different from Nucb2 physiological roles of nesfatin-3 and different impacts on the functioning of the tissues and on metabolism and its control. Our results clearly demonstrated that nesfatin-3 possessed divalent metal ion binding properties, which remained hidden in the nucleobindin-2 precursor protein.

**Supplementary Information:**

The online version contains supplementary material available at 10.1186/s12964-023-01181-6.

## Highlights

In the apo form, nesfatin-3 exists in solution as an extended molecule

Metal ion binding switches the conformation of the nesfatin-3 to more compact

Zn^2+^ ions exert a pronounced effect on the structure of nesfatin-3

The affinity of nesfatin-3 toward Ca^2+^ and Zn^2+^ increases compared to that of Nucb2

Video Abstract

**Supplementary Information:**

The online version contains supplementary material available at 10.1186/s12964-023-01181-6.

## Background

**Fig. 1:**
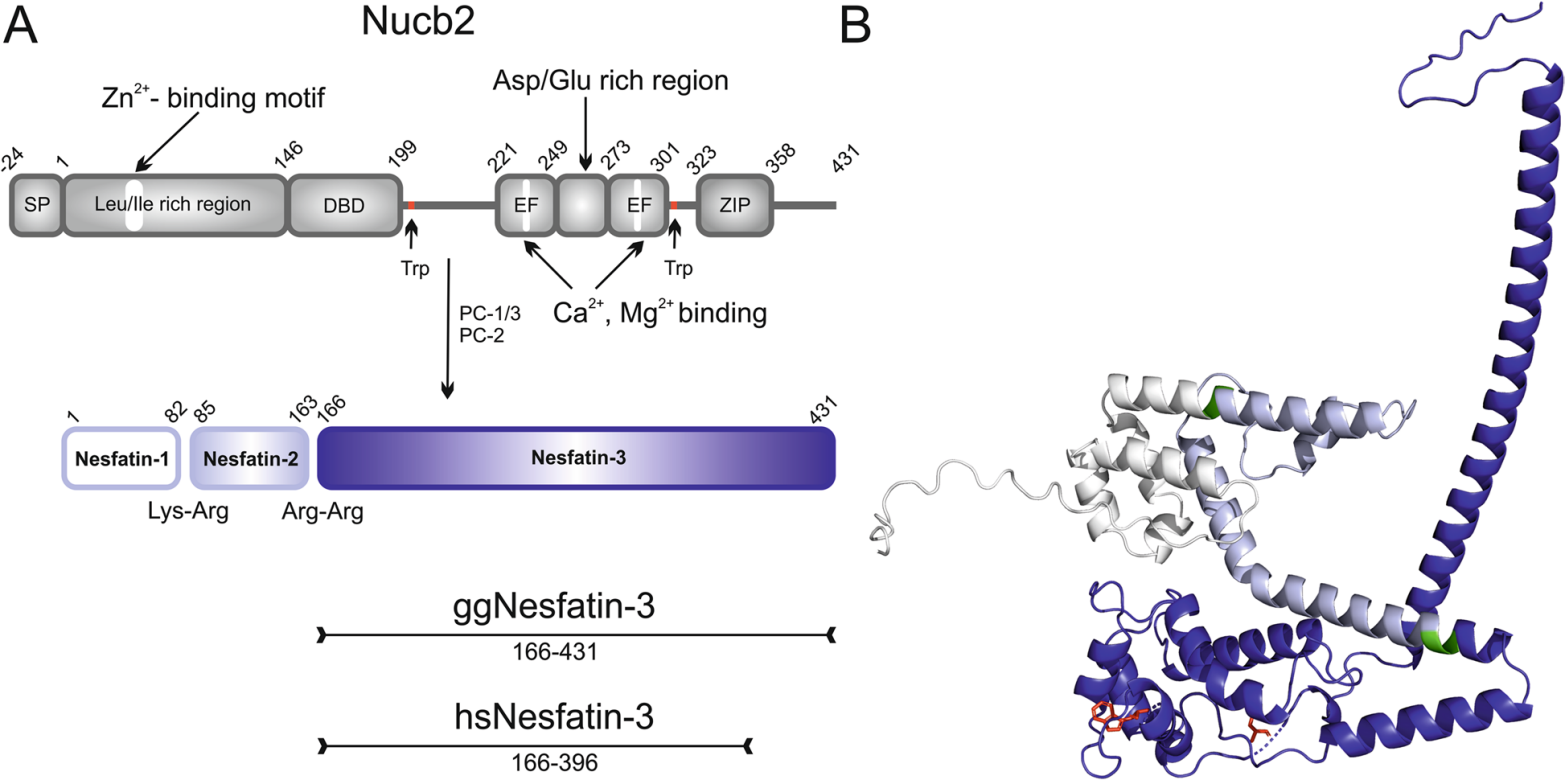
Illustration of a schematic domain representation and a predicted 3D structure of Nucb2. **A** Schematic representation of the domain structure of Nucb2 homologs and their proteolytic conversion. EF – EF-hand domain. ZIP – the leucine zipper motif. **B** The structure image of *Homo sapiens* Nucb2 from the UniProt Knowledgebase predicted by AlphaFold [3, 4] (AF-P80303-F1). Nesfatins are presented in white, light blue, and navy, respectively. PC cleavage sites are in green. Two Trp residues are in red

Multidomain proteins have been shown to be crucial to numerous signaling pathways acting as hubs facilitating signal integration and transduction. This is certainly the case for nucleobindin-2 (Nucb2). Nucb2 is a 396-amino acid precursor protein (Fig. 1A) composed of six domains: the Leu/Ile-rich region, the DNA-binding domain (DBD), two EF-hand domains, the acidic amino acid rich region, and the leucine zipper motif [1]. The leucine zipper domain is followed by a C-terminal intrinsically disordered region (IDR) of unknown function. After processing of the full-length Nucb2 by the specific prohormone convertases (PCs) PC1/3 and PC2 [2], three peptide products (Fig. 1A) can be derived. They have been named nesfatin-1 (amino acids 1–82), nesfatin-2 (aa 85–163) and nesfatin-3 (aa 166–396). Because Nucb2 and nesfatin-1 are colocalized in each tissue, their expression is often analyzed together as Nucb2. Additionally, of these three peptides, only nesfatin-1 exhibits some identified biological activity, while no defined functions have been ascribed thus far to nesfatin-2 and nesfatin-3.

Nucb2 was originally identified in several regions of the hypothalamus, such as the paraventricular nucleus, supraoptic nucleus, lateral hypothalamic area, and arcuate nucleus, and was found to be involved in feeding regulation [2, 5]. However, more recent studies have redefined and extended the function of Nucb2 far beyond its initially observed role as a negative modulator of food intake. The relevance of the metabolic function of Nucb2/nesfatin-1 has recently been suggested by its expression in adipocytes [6], reproductive organs [7, 8], and heart [9]. Circulating Nucb2 has been associated with panic disorder [10], reported depression [11], depression associated with subclinical hypothyroidism [12], and carcinogenesis [13] and correlated with inflammatory markers such as IL-6 and C-reactive protein as well as corticosterone [14]. Nucb2 was also recently linked to decreasing the motivational and rewarding value of food [15]. It is certainly easily perceived that the role of Nucb2 has been broadly studied. However, much less attention has been given to the molecular structure of this protein itself and the molecular details of its activity. Our group is already involved in the investigation of the structure–function relationship of Nucb2. All of our previous research data demonstrated that Nucb2 is a partially disordered protein with a structure divided into two parts: a mosaic-like N-terminal half consisting of disordered and ordered regions that alternately intertwine with each other and an unstructured C-terminal half. The predicted 3D structure of Nucb2 (Fig. 1B) authenticates this experimentally based model only to some degree. In addition, we observed that the secondary/tertiary structure of the protein changes upon binding Ca^2+^ and that Nucb2 undergoes Ca^2+^-dependent compaction as well and forms a mosaic-like structure at its C-terminal half [16]. We have also investigated the impact of two other ligands of Nucb2, Mg^2+^ and Zn^2+^ [17], which eventually allowed us to show that the Nucb2 protein was divided into two parts: the Zn^2+^-sensitive N-terminal half (encompassing nesfatin-1 and -2) and the Ca^2+^-sensitive C-terminal half (i.e., nesfatin-3). Recently, to obtain broader insight into the structure of Nucb2, we decided to take a closer look at the molecular properties of the proteolytical products of Nucb2 conversion, i.e., nesfatin-1 and nesfatin-2, as well as nesfatin-1 coupled with nesfatin-2 in a head-to-tail manner (nesfatin-1/2, encompassing the N-terminal half of the Nucb2 molecule). We demonstrated that apo-nesfatin-1 displays a highly disordered structure that, upon Zn^2+^ treatment, undergoes a disorder-to-order transition accompanied by dimerization of the peptide and the formation of the hydrophobic core [18]. In contrast, the binding of Zn^2+^ ions by ordered nesfatin-2 and, surprisingly, by nesfatin-1/2 strongly destabilized the structures of both proteins. Subsequently, a more detailed investigation revealed that structurally distinct nesfatin-1 and nesfatin-2 are rather interdependent when linked together, as indicated by the observed properties of nesfatin-1/2, which in turn were not a simple sum of the properties exhibited by the separated peptides [18]. These results have raised the intriguing conclusion that nesfatins released by PC cleavage may have completely different molecular properties than Nucb2 and hence may reveal distinctive physiological functions. To complete the structural analysis of Nucb2 and give some new hints of the unique features of this precursor protein and its isolated peptides, one piece of the puzzle was still missing—nesfatin-3. While published experimental results concentrate on either Nucb2 or nesfatin-1, the detailed analysis of nesfatin-3 has been left a subject of speculation. To address the questions regarding the structure of nesfatin-3 and to determine to what extent the structure of the C-terminus of Nucb2 resembles that of neafstin-3, the present study aimed to investigate the molecular properties of this peptide in its apo form and in its form bound to different metal ions through multiple analytical techniques.

In this work, we present the first structural characterization of the nesfatin-3 protein. The study was designed as a comparative analysis of the molecular properties of two homologs (based on the BLAST algorithm, both proteins share 85% of amino acid identity), nesfatin-3 from *Homo sapiens* (hsNesfatin-3) and from *Gallus gallus* (ggNesfatin-3), in the absence and presence of Mg^2+^, Ca^2+^ and Zn^2+^ ions, natural ligands of Nucb2. Our results show that both nesfatin-3s were abundant in α-helical structures and disordered regions. They both interacted with divalent metal ions, and this interaction manifested itself in a conformational change leading to the compaction of the protein molecules. However, the influence of each metal cation differed from each other, and the final effect exerted on the protein molecule varied between homologs. While hsNesfatin-3 existed in solution as a dimer, regardless of the presence of the ions, ggNesfatin-3 formed mixtures of different percentages of monomeric and dimeric species. The addition of Mg^2+^ enhanced the dimerization of ggNesfatin-3. Our analysis revealed that ggNesfatin-3 possessed two Ca^2+^-, two Mg^2+^-, and three Zn^2+^-binding sites, whereas hsNesfatin-3 bound two ions of Ca^2+^ and one of Zn^2+^. Although we expected that the molecules of nesfatin-3s may differ from each other, surprisingly, the results obtained during recent studies showed them as entirely different compared even to the structures of the nucleobindin-2 precursors [16, 17]. The changes affected the secondary, tertiary, and quaternary structures. While both Nucb2s were characterized by low affinity toward Mg^2+^, isolated ggNesfatin-3 possessed a much higher affinity for these cations. The affinity for Zn^2+^ was also dramatically increased after the removal of the nesfatin1/2-encompassing sequences, which was concluded from a reduction in *K*_d_ from micro- to nanomolar values. The observed alterations suggest different physiological roles of nesfatin-3 in Nucb2 and different impacts on the functioning of the tissues and on metabolism and its control. These results clearly demonstrate that nesfatin-3 possesses divalent metal ion binding properties, which remain hidden in the nucleobindin-2 precursor protein.

## Methods

### Protein preparation

The cDNAs of the nesfatin-3s were subcloned into the pQE-80L/HRV3C vector using standard DNA manipulation procedures as described in a previous report [17]. hsNesfatin-3 was expressed and purified as reported previously [16, 17]. The procedure used for ggNesfatin-3 was slightly modified. The cleavage of His-tagged ggNesfatin-3 was carried out on Ni–NTA resin, after which the eluate was concentrated and transferred to 50 mM NaH_2_PO_4_ × 2H_2_O (pH 7.0), 700 mM (NH_4_)_2_SO_4_ using Amicon Ultra-4 centrifugal filters (Merck Millipore Ltd.). The sample containing ggNesfatin-3 was then subjected to hydrophobic interaction chromatography on a HiTrap Phenyl HP 5 ml column (Cytiva) connected to the ÄKTA Avant FPLC system (GE Healthcare). Samples were eluted using a decreasing salt gradient of (NH_4_)_2_SO_4_ (0.7–0 M) for 15 min. Selected fractions of ggNesfatin-3 were concentrated and desalted to either 20 mM Tris (pH 7.5), 150 mM NaCl or 20 mM Hepes (pH 7.5), 150 mM NaCl on a HiTrap Desalting Column. The protein concentrations were measured at 280 nm utilizing the following extinction coefficients: ggNesfatin-3—13,980 M^−1^ · cm^−1^ and hsNesfatin-3—16,960 M^−1^ · cm^−1^, estimated from the amino acid sequence according to Gill and von Hippel [19]. The chemical identity of the targeted proteins was evaluated by mass spectrometry.

### Circular dichroism (CD) spectroscopy

Far-UV CD spectra were recorded using a JASCO J-815 spectropolarimeter (Jasco Inc.) equipped with a Peltier-type temperature controller (CDF-426S/15, Jasco Inc.). The spectra were collected in a range of 195–270 nm with a scanning speed of 50 nm/min at 20 °C in a 100QS 1-mm path length cuvette (Hellma). The data pitch was 0.5 nm, and the final spectrum was obtained after averaging three measurements. The spectra were recorded for 10 µM nesfatin-3 in 20 mM Tris (pH 7.5), 150 mM NaCl supplemented with EDTA (5 mM), MgCl_2_ (10 mM), CaCl_2_ (0.01–10 mM) or ZnCl_2_ (0.025–0.8 mM). Secondary structure composition was estimated using CDNN software (version 2.1) [20].

### Limited proteolysis

Both ggNesfatin-3 and hsNesfatin-3 (0.5 mg/ml) were digested with endopeptidase Glu-C (1:5000 w/w) in 20 mM Tris (pH 7.5), 150 mM NaCl supplemented with EDTA (5 mM), MgCl_2_ (0–10 mM), CaCl_2_ (0–10 mM) or ZnCl_2_ (0–0.8 mM). The digestion reactions were performed at 20 °C for 0–24 h (hsNesfatin-3) and 0–240 min (ggNesfatin-3). Aliquots of 5 µg (10 µl) were removed at appropriate time points, and the reaction was stopped by adding SDS loading buffer. The progress of the reaction was analyzed by SDS‒PAGE.

### Fluorescence spectroscopy

Steady-state fluorescence was monitored at 20 °C in a Fluorolog-3 spectrofluorometer (Horiba Jobin Yvon Inc.). Emission spectra for aromatic chromophores were recorded for 3 µM nesfatin-3s in 20 mM Tris (pH 7.5), 150 mM NaCl supplemented with EDTA (5 mM), MgCl_2_ (10 mM), CaCl_2_ (0.01–10 mM) or ZnCl_2_ (0.025–0.8 mM). The spectra were recorded between 310–500 nm, with an excitation wavelength of 295 nm, immediately after the metal stocks were added (especially for the Zn^2+^-bound nesfatins). Measurements were performed in a 0.3-cm path-length quartz cuvette (Hellma) using an integration time of 0.5 s and an increment of 1 nm. Slits with bandwidths of 3.0 nm were used for both the excitation and emission channels. The obtained spectra were corrected for the buffer contribution.

### Sedimentation velocity analytical centrifugation (SV-AUC)

Sedimentation-velocity analytical ultracentrifugation (SV-AUC) was conducted on a Beckmann Coulter Proteome-Lab XL-I ultracentrifuge (software version 6.0) equipped with a 4-position An-60Ti rotor. Protein samples (0.50 mg/ml, 0.75 mg/ml, and 1.0 mg/ml) were prepared in 20 mM Tris (pH 7.5), 150 mM NaCl supplemented with EDTA (5 mM), MgCl_2_ (10 mM), CaCl_2_ (10 mM) or ZnCl_2_ (0.1–0.8 mM). All samples were subjected to centrifugation into 2-channel carbon-epoxy cells with 12-mm optical pathlengths at 20 °C and 50 000 rpm using a step size of 0.003 cm and a delay time of 0 s. The absorbance across the cell was measured at 280 nm. The proteins’ partial specific volumes (0.72389 and 0.7273 ml/g for ggNesfatin-3 and hsNesfatin-3, respectively), buffer density (1.0059 g/ml, 1.0057 g/ml, and 1.0060 g/ml for EDTA-, MgCl_2_- or CaCl_2_/ZnCl_2_-containing buffer, respectively) and viscosity (1.0265 mPa · s, 1.0258 mPa · s and 1.0228 mPa · s for EDTA-, MgCl_2_- or CaCl_2_/ZnCl_2_-containing buffer, respectively) were calculated with SEDNTERP [21]. Time-corrected data [22] were analyzed with SEDFIT software (version 16.1c) using the built-in continuous sedimentation coefficient distribution model, *c*(s). Maximum-entropy regularization of the *c*(s) models was set to a confidence level of 0.68 [23, 24]. The fits were calculated alternately using simplex and Marquardt–Levenberg algorithms. The obtained sedimentation coefficients were standardized *s*_20,w_ values based on the solute and buffer properties. Data, fits, residuals, and *c*(s) distributions were plotted using the Gussi interface (version 1.4.2) [25].

### Isothermal titration calorimetry (ITC)

The binding of Mg^2+^, Ca^2+^, and Zn^2+^ to nesfatin-3s was monitored with a Nano-ITC calorimeter (TA Waters, USA) at 25 °C with a Hastelloy cell volume of 1 ml in overflow mode. All experiments were performed in 20 mM Hepes (pH 7.5), 150 mM NaCl. The protein (titrate) concentration was in the range of 95–120 μM, and the metal ion (titrant) concentration was 2 mM. Concentrations of titrate and titrant were adjusted to obtain the best isotherms for proper analysis of equilibria and to highlight different processes throughout the titration experiment. Titration experiments were performed after initial system equilibration to a medium setting of a slope of 0.3 µW × hr^−1^ and 0.03 µW standard error by sequential injections of 5.22 μl of the titrant into the cell at 300-s intervals and stirring at 250 rpm. The control titrations were performed with an identical setup but without the protein. From the experimental isotherms, control isotherms were subtracted to obtain the net reaction heat. The titration data were analyzed using NanoAnalyze (version 3.11.0) by integrating data and subtraction of both baseline and dilution heat. Preprocessed data were fitted to different models supplied in NanoAnalyze, such as one-site, sequential binding, and multiple-site models, depending on the titration. Thermodynamic parameters, i.e., dissociation constant (*K*_d_), binding stoichiometry (n), enthalpy change (Δ*H*), and entropy change (Δ*S*), were calculated by curve fitting of the model to the binding isotherms. The error analysis was carried out by a Monte Carlo approach with at least 1000 trials and a 0.95 level of confidence.

## Results

### Effect of metal ion binding on the far-UV CD spectra of nesfatin-3s

**Fig. 2:**
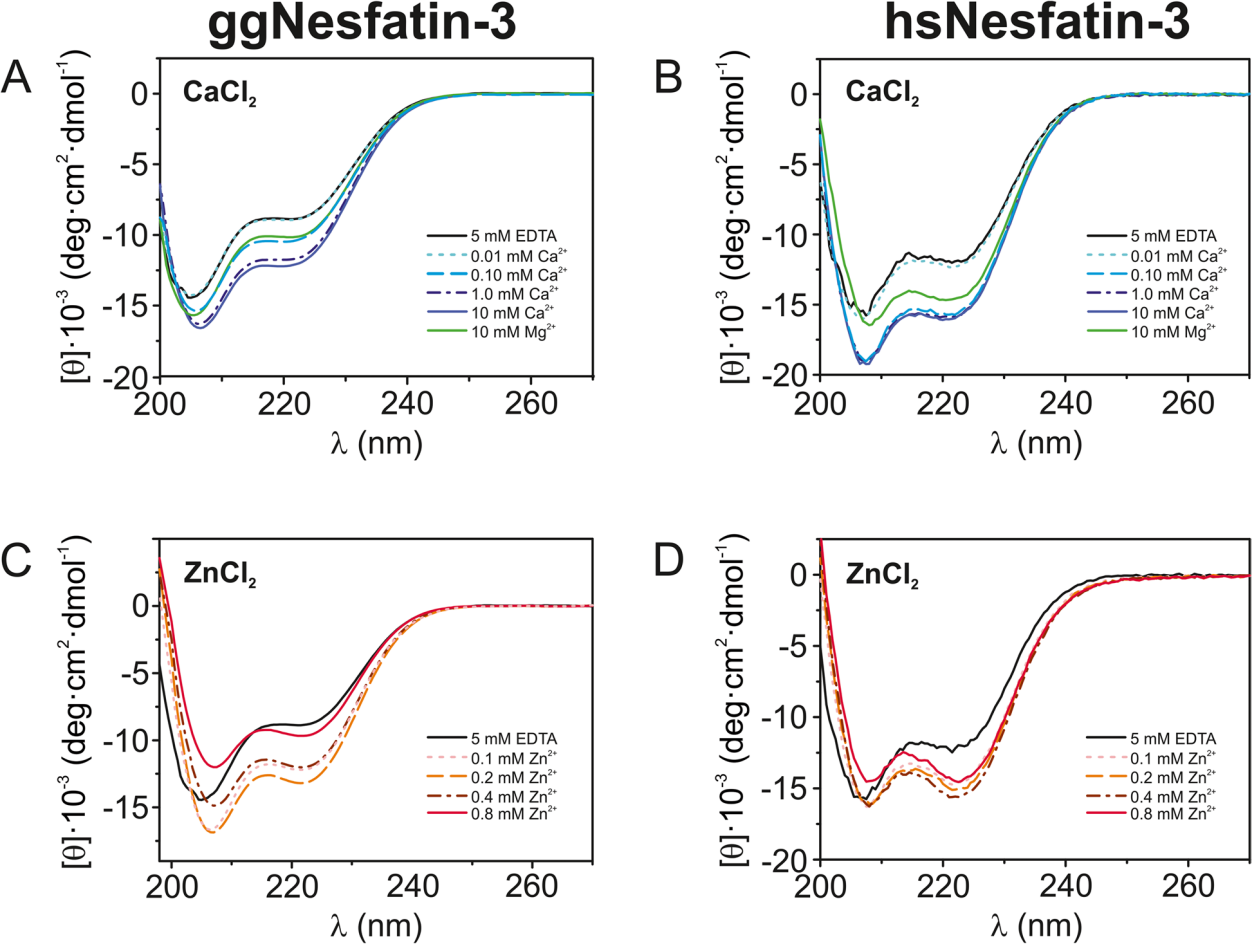
Far-UV CD spectra of ggNesfatin-3 (**A**, **C**) and hsNesfatin-3 (**B**, **D**) recorded in the presence of either Ca^2+^ (**A**–**B**) or Zn^2+^ (**C**–**D**) ions. For clarity, only selected curves are shown, whereas the complete set of data is presented in Table 1. The spectra were recorded for 10 µM nesfatin-3s. The data shown are representative of three independent experiments

The spectra of nesfatin-3s measured in the absence and presence of various divalent metal ions are presented in Fig. 2. In all cases, the spectra showed features typical of the α-helical conformation, with two negative maxima at 208 nm and 222 nm [26]. Significant differences were observed in metal ion titration, suggesting specific binding interactions between the proteins and the metal ions. The deconvolution of CD data with CDNN software [20] revealed that both apo-nesfatin-3s were abundant in α-helices (ggNesfatin-3: 32 ± 1.3%, hsNesfatin-3: 32 ± 1.3%; Table 1) and had a significant amount of random coil regions (ggNesfatin-3: 33 ± 1.0%; hsNesfatin-3: 28 ± 0.3%). Further analysis of the far-UV CD spectra revealed that the binding of Mg^2+^ ions to the nesfatin-3s induced an observable modification of the secondary structure of both proteins, leading to a moderate increase in the α-helical structure (to 38 ± 1.0% and 38 ± 2.7% for ggNesfatin-3 and hsNesfatin-3, respectively) with a simultaneous decrease in random coils (to 31 ± 0.6% and 26 ± 1.4% for ggNesfatin-3 and hsNesfatin-3, respectively). Even more pronounced negative changes were observed when saturating concentrations of Ca^2+^ ions (0–10 mM) were added to the protein mixtures (Fig. 2A–B). Both nesfatin-3s were sensitive to Ca^2+^, as some structural rearrangements were observed even at very low Ca^2+^ concentrations (0.025 mM for ggNesfatin-3 and 0.01 mM for hsNesfatin-3) (Table 1). Figure 2C–D also shows the series of spectra of Zn^2+^-bound nesfatin-3s, for which the variation in the relative intensity of the negative CD bands was quite significant. Similarly, nesfatin-3s maintained a comparably ordered secondary structure dominated by α-helices following the binding of Zn^2+^. However, only the presence of lower concentrations of Zn^2+^ (ggNesfatin-3 – up to 0.2 mM, hsNesfatin-3 – to 0.4 mM) induced a systematic increase in α-helix content with a simultaneous decrease in random coils (Table 1), reflecting a conformational change. At higher Zn^2+^ concentrations, we also observed some rearrangements of the secondary structures of nesfatin-3s, especially compared to those of the apo forms. Surprisingly, an unexpected reduction in the amplitude of the signal was also observed (Fig. 2C–D). We did not notice, however, a concomitant precipitation of the proteins. Taken together, the far-UV CD spectra suggest that the analyzed neaftin-3 s exist in solution as moderately structured proteins with the propensity for further structurization following the binding of the divalent metal ions. It is likely that the structures of both proteins undergo some additional changes reflecting the loss of the CD signal in the presence of high Zn^2+^ concentrations.

**Table 1:** Secondary structure composition of ggNesfatin-3 and hsNesfatin-3 in the presence of various metal ions. The deconvolution of CD data was performed using CDNN [20]. The table presents the percentage of each structural element in the secondary structure. The data are the average of three independent experiments

Protein	Agent	Concentration (mM)	α-helix (%)	Antiparallel β-sheet (%)	Parallel β-sheet (%)	β-turn (%)	Random coil (%)
ggNesfatin-3	EDTA	5	32 ± 1.3	8 ± 0.3	9 ± 0.4	17 ± 0.2	33 ± 1.0
	MgCl_2_	10.00	38 ± 1.0	7 ± 0.2	8 ± 0.2	16 ± 0.1	31 ± 0.6
		0.010	35 ± 0.6	8 ± 0.1	9 ± 0.2	16 ± 0.1	32 ± 0.5
		0.025	34 ± 0.1	8 ± 0.0	9 ± 0.1	17 ± 0.0	32 ± 0.1
		0.050	38 ± 1.0	7 ± 0.2	8 ± 0.2	16 ± 0.2	30 ± 0.6
	CaCl_2_	0.075	39 ± 1.5	7 ± 0.2	8 ± 0.3	16 ± 0.2	30 ± 1.0
		0.100	40 ± 0.4	7 ± 0.0	8 ± 0.1	16 ± 0.1	29 ± 0.2
		0.250	44 ± 2.3	6 ± 0.3	7 ± 0.5	16 ± 0.3	27 ± 1.4
		0.500	44 ± 0.2	6 ± 0.0	7 ± 0.1	16 ± 0.0	27 ± 0.1
		0.750	44 ± 0.0	6 ± 0.2	7 ± 0.2	16 ± 0.2	26 ± 0.8
		1.000	44 ± 0.5	6 ± 0.1	7 ± 0.1	15 ± 0.1	28 ± 0.3
		10.00	45 ± 3.0	6 ± 0.5	7 ± 0.6	15 ± 0.4	27 ± 1.7
		0.050	45 ± 1.1	6 ± 0.2	7 ± 0.2	15 ± 0.2	27 ± 0.6
		0.100	45 ± 0.5	6 ± 0.1	7 ± 0.1	15 ± 0.1	27 ± 0.3
	ZnCl_2_	0.200	47 ± 1.0	6 ± 0.1	7 ± 0.2	15 ± 0.1	25 ± 0.6
		0.300	46 ± 0.8	6 ± 0.1	7 ± 0.1	15 ± 0.1	26 ± 0.4
		0.400	44 ± 1.6	6 ± 0.2	7 ± 0.3	15 ± 0.2	26 ± 1.0
		0.500	43 ± 0.4	6 ± 0.1	7 ± 0.1	15 ± 0.1	27 ± 0.2
		0.600	41 ± 0.6	7 ± 0.1	7 ± 0.1	16 ± 0.1	29 ± 0.4
		0.800	33 ± 0.7	8 ± 0.2	9 ± 0.2	17 ± 0.1	33 ± 0.5
hsNesfatin-3	EDTA	5	32 ± 1.3	14 ± 0.8	8 ± 0.2	18 ± 0.1	28 ± 0.3
	MgCl_2_	10.00	38 ± 2.7	10 ± 0.8	8 ± 0.4	18 ± 0.1	26 ± 1.4
		0.010	39 ± 0.7	11 ± 0.3	7 ± 0.1	18 ± 0.0	25 ± 0.2
	CaCl_2_	0.025	42 ± 0.9	9 ± 0.3	7 ± 0.1	18 ± 0.1	24 ± 0.3
		0.050	48 ± 0.2	7 ± 0.1	6 ± 0.1	17 ± 0.1	22 ± 0.1
		0.075	49 ± 1.3	7 ± 0.4	6 ± 0.2	17 ± 0.1	21 ± 0.6
		0.100	49 ± 2.7	7 ± 0.8	6 ± 0.3	17 ± 0.4	21 ± 1.1
		0.250	49 ± 0.9	7 ± 0.4	6 ± 0.1	17 ± 0.4	21 ± 0.3
		0.500	49 ± 2.5	7 ± 0.7	6 ± 0.4	17 ± 0.4	21 ± 1.0
		0.750	52 ± 3.7	6 ± 1.1	6 ± 0.6	17 ± 0.6	20 ± 1.5
		1.000	50 ± 1.9	6 ± 0.5	6 ± 0.3	17 ± 0.3	21 ± 0.9
		10.00	51 ± 0.6	6 ± 0.1	6 ± 0.1	17 ± 0.1	21 ± 0.3
		0.050	43 ± 0.3	8 ± 0.1	7 ± 0.1	17 ± 0.0	25 ± 0.1
		0.100	44 ± 0.1	7 ± 0.1	7 ± 0.1	17 ± 0.0	25 ± 0.2
		0.200	45 ± 1.6	7 ± 0.5	7 ± 0.3	17 ± 0.3	25 ± 0.7
	ZnCl_2_	0.300	44 ± 0.2	7 ± 0.1	7 ± 0.0	17 ± 0.0	26 ± 0.1
		0.400	45 ± 1.5	6 ± 0.4	7 ± 0.3	16 ± 0.2	26 ± 0.6
		0.500	43 ± 1.2	7 ± 0.3	7 ± 0.2	16 ± 0.1	27 ± 0.6
		0.600	44 ± 1.9	7 ± 0.6	7 ± 0.3	16 ± 0.3	26 ± 0.9
		0.800	43 ± 1.4	7 ± 0.3	7 ± 0.2	16 ± 0.1	27 ± 0.7

### Effect of metal ions on the tertiary structure of nesfatin-3 analyzed with limited proteolysis

**Fig. 3:**
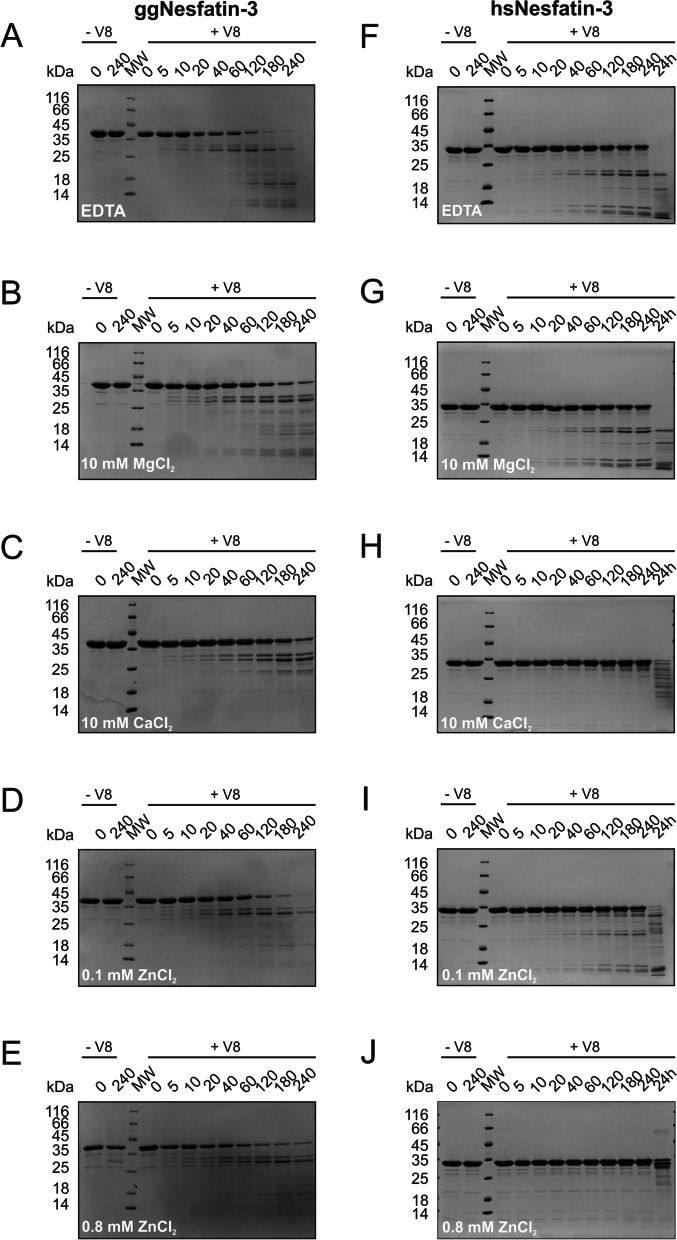
SDS‒PAGE analysis of limited proteolysis products at a range of digestion times (expressed in minutes) indicated on the top of each panel (up to 240 min for ggNesfatin-3 and 24 h for hsNesfatin-3). Both ggNesfatin-3 (**A**–**E**) and hsNesfatin-3 (**F**–**J**) were incubated with V8 protease at 20 °C and at an enzyme-to-substrate ratio of 1:5000 in the absence (**A**, **F**) and presence of 10 mM MgCl_2_ (**B**, **G**), 10 mM CaCl_2_ (**C**, **H**), 0.1 mM ZnCl_2_ (**D**, **I**) and 0.8 mM ZnCl_2_ (**E**, **J**). MW – molecular mass marker; -V8 – control reactions with no protease

**Fig. 4:**
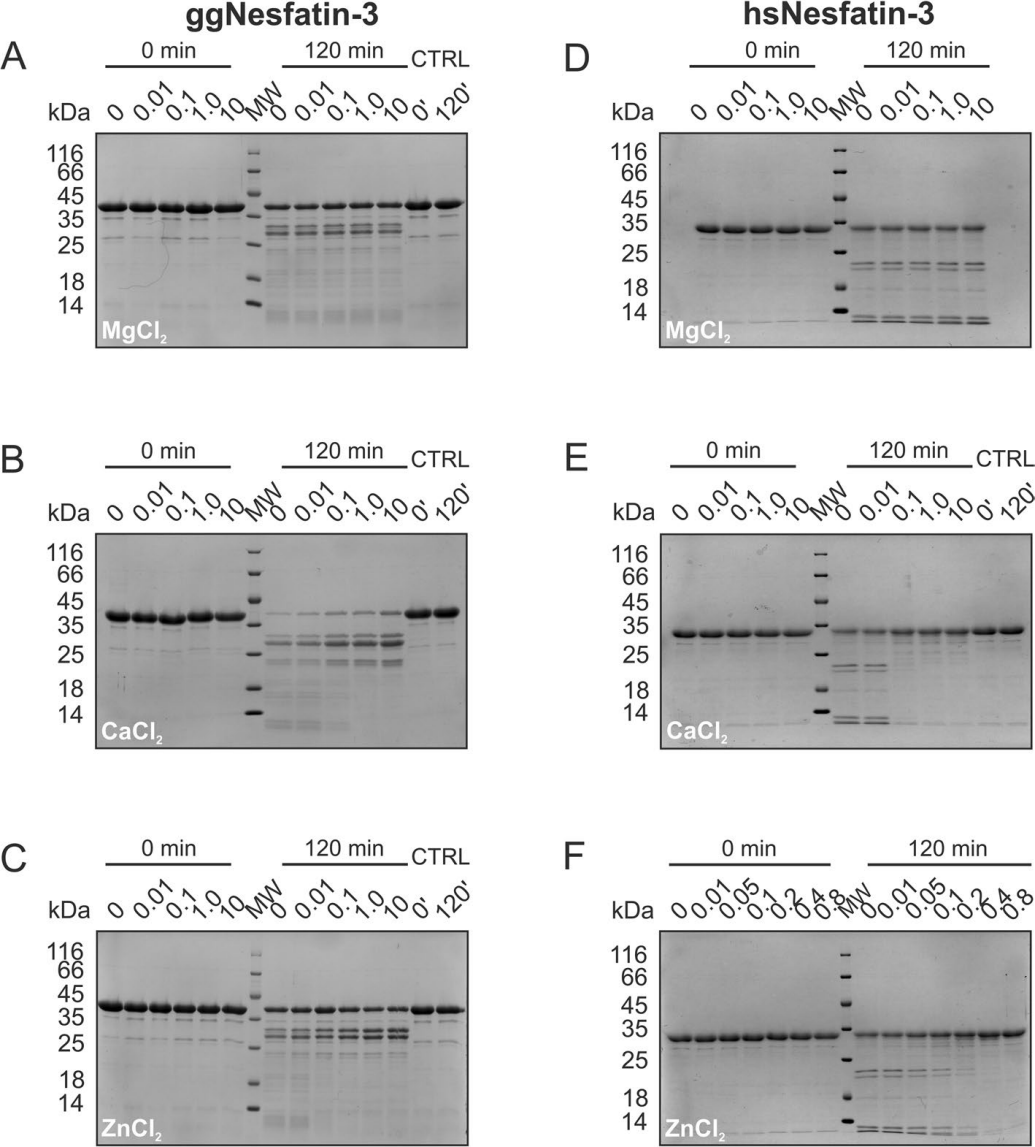
SDS‒PAGE analysis of limited proteolysis products at different Mg^2+^ (**A**, **D**), Ca^2+^ (**B**, **E**), and Zn^2+^ (**C**, **F**) concentrations (expressed in mM) indicated on the top of each panel. Both ggNesfatin-3 (**A**–**C**) and hsNesfatin-3 (**D**–**F**) were incubated with V8 protease at 20 °C at an enzyme-to-substrate ratio of 1:5000. MW – molecular mass marker; CTRL – control reactions with no protease

The far-UV CD spectra showed that the secondary structures of ggNesfatin-3 and hsNesfatin-3 are sensitive to divalent metal ions. In the presence of Mg^2+^, Ca^2+^, and Zn^2+^ ions, structurization to a more helical state was observed for both homologs. To further investigate the structural changes in nesfatin-3s, we utilized a limited proteolysis experiment. To monitor the putative cation-induced conformational transition of the protein tertiary structure, glutamyl endopeptidase Glu-C (V8) from *Staphylococcus aureus* was used. The V8 protease cleaves peptide bonds on the C-terminal part of Asp and Glu amino acid residues [27]. Digestion occurs especially at structurally unordered sites with enhanced flexibility [28]. Figure 3 shows the results of SDS–PAGE analyses of the proteolytic fragments of nesfatin-3s obtained without or with metal ions added to the reaction mixtures. Under the conditions used, the reaction for ggNesfatin-3 seemed to proceed rather quickly in the absence of metal ions, so that after 4 h of incubation, apo-ggNesfatin-3 was fully digested (Fig. 3A), yielding the formation of a main product of approx. 27 kDa as well as lower molecular weight peptides. In contrast to the results obtained from CD spectroscopy, the resulting digestion profile of Mg^2+^-saturated ggNesfatin-3 turned out to be nearly the same (albeit slightly slower) compared to the pattern obtained in the absence of metal ions (compare Fig. 3B and Fig. 3A), regardless of the concentration of Mg^2+^ used (Fig. 4A). On the other hand, after the formation of mostly helical Ca^2+^-ggNesfatin-3 (Table 1), the digestion appeared to be greatly retarded, with the full-length protein still present in the reaction mixture (Fig. 3C) after 4 h, especially when the concentration of Ca^2+^ rose above 0.1 mM (Fig. 4B). Unlike Mg^2+^- or Ca^2+^-bound ggNesfatin-3, Zn^2+^-saturated intact protein was barely to moderately present in the reaction mixture, indicating almost complete digestion (Fig. 3D, E). In the presence of lower Zn^2+^ concentrations (Fig. 4C), the digestion of ggNesfatin-3 resulted in a more or less similar pattern as that in the presence of Mg^2+^, with the characteristic accumulation of a double band with a molecular mass of approximately 27 kDa.

Under the same conditions, digestion of apo-hsNesfatin-3 was much slower and resulted in the presence of a significant amount of the intact full-length protein in the reaction mixture together with two major peptide products of approx. 20 kDa (Fig. 3F) and some low molecular mass fragments. The addition of Mg^2+^ did not change the profile of the digestion (Figs. 3G and 4D). Concomitantly, Ca^2+^-bound hsNesfatin-3 appeared to be even more resistant to proteolysis when reacted under identical conditions, and even after the reaction proceeded for 24 h, the protein band was still seen in trace amounts in the stained SDS − PAGE gel (Fig. 3H). The critical concentration of Ca^2+^, at which the transition from less to highly stable protein occurred, was found to be somewhere between 0.01–0.1 mM (Fig. 4E). The addition of 0.1 mM Zn^2+^ promoted the conformation that was in between those observed previously for Mg^2+^/Ca^2+^-saturated protein (Fig. 3I), whereas the addition of 0.8 mM Zn^2+^ made the protein relatively insensitive to proteolytic cleavage (Figs. 3J and 4F).

Eventually, we concluded that the proteolysis data presented here appeared to indicate that holo-nesfatin-3s gain some (likely diverse) new structural features that render regions of the polypeptide chains resistant to proteolysis. One possible explanation for such a hypothesis is that metal cation-saturated ggNesfatin-3 and hsNesfatin-3 possess quite different tertiary/quaternary structures rather than different secondary structures. In the next experiments, we tested this hypothesis.

### Tertiary structure changes in nesfatin-3 induced by divalent metal ions analyzed with fluorescence spectroscopy

**Fig. 5:**
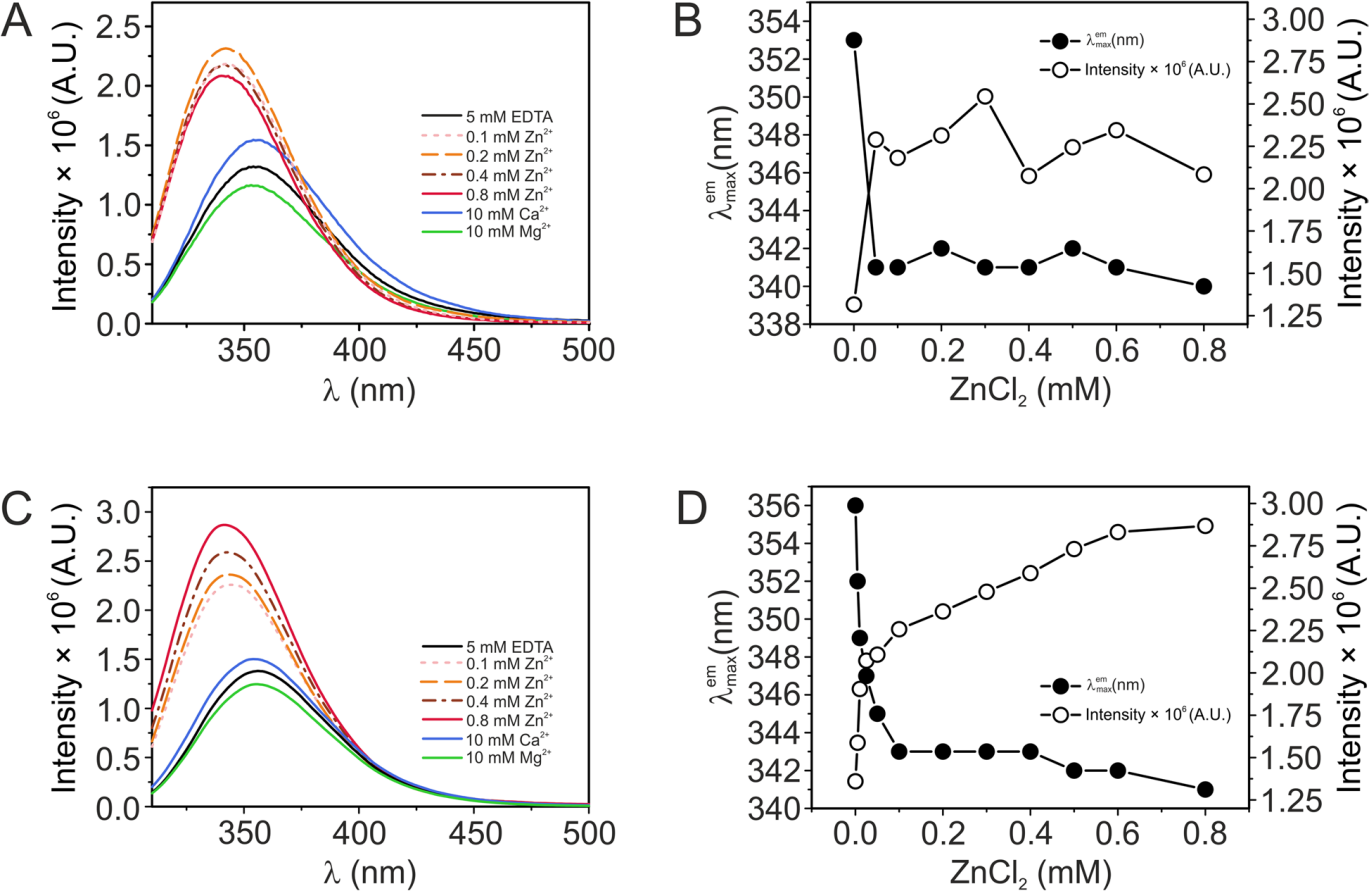
Intrinsic fluorescence spectra of Nucb2s in the presence of 5 mM EDTA, 10 mM Mg^2+^, and 10 mM Ca^2+^ and in the presence of increasing concentrations of Zn^2+^ (100–800 µM). Panels show the fluorescence spectra of ggNesfatin-3 (**A**–**B**) and hsNesfatin-3 (**C**–**D**). For clarity, only selected curves are shown (**A**, **C**). The protein concentration was 3 µM. The measurements were performed at 20 °C

The effect of divalent metal ion binding on the tertiary structure of nesfatin-3 was next investigated by tryptophan fluorescence analyses. The photophysical properties of Trp residues are highly sensitive to their microenvironment [29, 30], which means that they may act as reporter groups for local conformation changes. Nesfatin-3 has two Trp residues: one preceding the first EF-hand domain and one following the second (Fig. 1B). Both residues appeared to be fully exposed to the solvent in the absence of metal ions, as emission maxima were observed at 353 nm and 356 nm for apo-ggNesfatin-3 and apo-hsNesfatin-3, respectively (Fig. 5). Analysis of the fluorescence spectra measured in the Mg^2+^-, Ca^2+^- and Zn^2+^-bound states revealed that the intensity increased moderately to significantly in the following order of Ca^2+^  > Zn^2+^, whereas for Mg^2+^-nesfatin-3s, we observed a decrease in fluorescence intensity (Fig. 5A, C). Notably, the presence of Mg^2+^ had no impact on the position of the Trp emission maxima of both nesfatin-3s. After Ca^2+^ binding, the fluorescence maxima occurred at 351 nm and 354 nm, with a moderate increase in the fluorescence intensity, showing that in the Ca^2+^-bound state, Trp residues were still present in a solvent-exposed environment (Fig. 5A, C). In contrast, the addition of 100–800 µM Zn^2+^ led to a visible blueshift of the fluorescence maxima of 13 nm for ggNesfatin-3 (Fig. 5B) and 15 nm for hsNesfatin-3 (to 340 nm and 341 nm for ggNesfatin-3 and hsNesfatin-3, respectively) (Fig. 5D), accompanied by a substantial increase in the fluorescence emission. Unfortunately, the fluorescence intensity of the Trp residues of ggNesfatin-3 in the presence of 800 µM Zn^2+^ consequently decreased. The hypsochromic shift in the fluorescence emission maximum demonstrated that the binding of Zn^2+^ led to a significant change in the local environment of the tryptophan residues to a relatively less polar environment (i.e., water-sequestered), indicating that Zn^2+^ binding induces substantial structural reorganization within the tertiary structure of the nesfatin-3 molecule.

### Quaternary structure changes in nesfatin-3 measured with SV-AUC

**Fig. 6:**
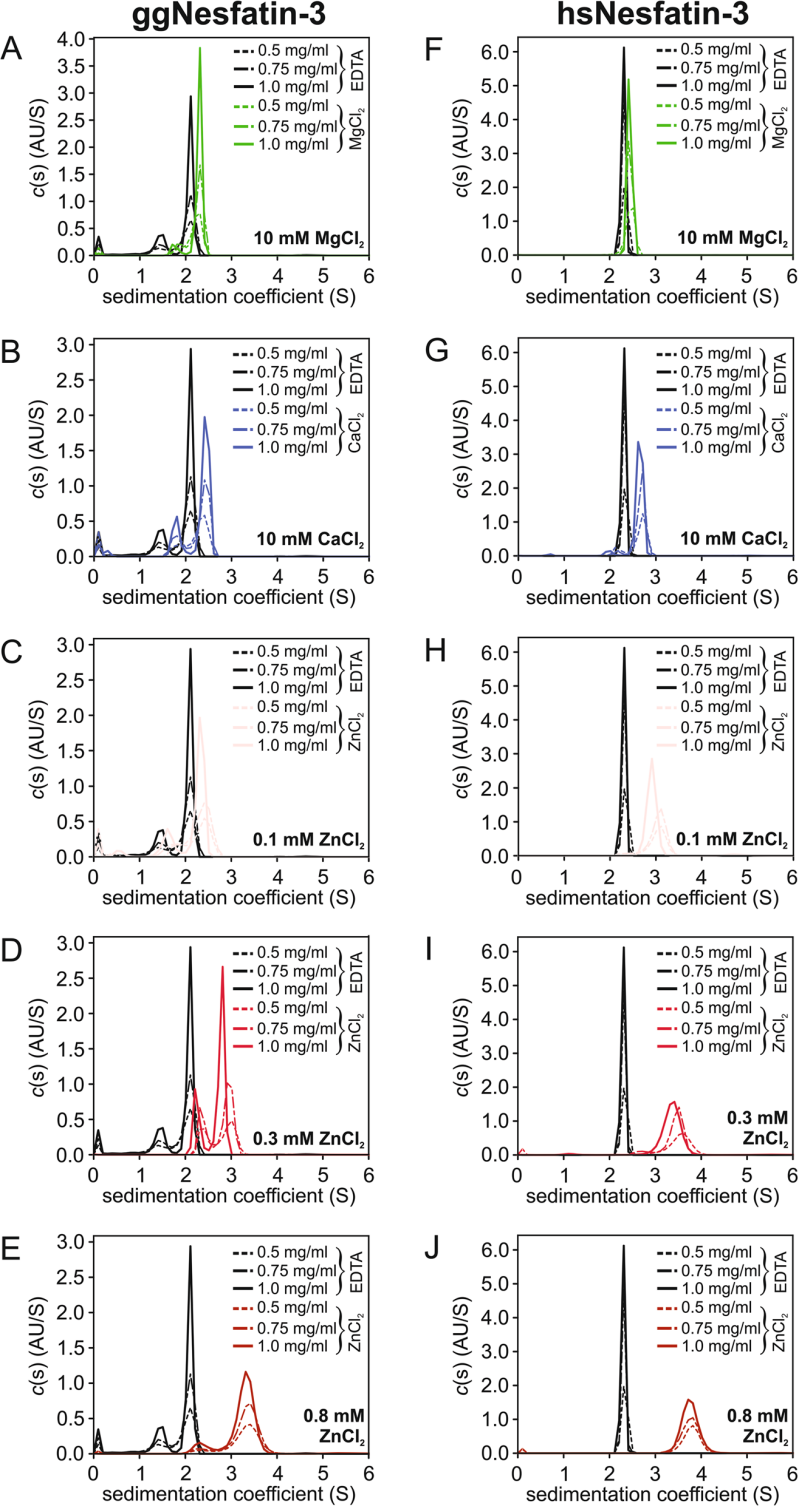
Size distribution characterization of the nesfatin-3s in the presence of Mg^2+^ (**A**, **F**), Ca^2+^ (**B**, **G**), and Zn^2+^ (**C**–**E**, **H**–**J**) ions. The SV-AUC measurements were performed for three protein concentrations (c): 0.5 mg/ml (dotted lines), 0.75 mg/ml (dash-dotted lines), and 1.0 mg/ml (solid lines). Both ggNesfatin-3 (**A**–**E**) and hsNesfatin-3 (**F**–**J**) were run at 50 000 rpm and 20 °C

Prompted by the above-described observations, the SV-AUC technique was used to study the solution behavior of the nesfatins. The experiments were run with three protein concentrations to provide broader data on sample composition and collect information on the observed species’ molecular weights and sedimentation coefficients and on the sample heterogeneity. Figure 6 shows all the sedimentation coefficient distributions obtained from the *c*(s) analysis method for the nesfatin-3s. In the absence of metal ions, the sedimentation profiles showed two intermediate states of apo-ggNesfatin-3 (Fig. 6A) corresponding to a mixture of a presumably monomeric (apparent molecular mass, MM_app_, of 26.1 kDa for the minor fraction, at the lowest protein concentration) and dimeric (43.8 kDa) species. Both components were consistently characterized by imprecise MM_app_ values, one of which shifted with the concentration progressively toward the MM value of the dimer. We explained this discrepancy by the fact that monomers and dimers are typically in a fast (relative to the time scale of the velocity experiment) equilibrium, which may be a reason for their rapid and reversible association. In the presence of Mg^2+^ (Fig. 6A), an increase in the population with a higher than monomeric molecular mass was observed (at 2.28–2.40 S), reflecting a positive effect of Mg^2+^ binding on the overall quaternary structure. Samples of 0.75 mg/ml and 1.0 mg/ml Mg^2+^-ggNesfatin-3 also contained a small portion of some lower molecular weight species that could have resulted from its degradation, suggesting that the binding of Mg^2+^ and/or conditions of the SV experiments may influence the stability of the dimeric form. Similarly, two populations of molecules with underestimated MMs between the monomeric and dimeric forms (Table 2) were observed for Ca^2+^-ggNesfatin-3 (Fig. 6B) and Zn^2+^-ggNesfatin-3 (Fig. 6C–E).

**Table 2:** Results of SV-AUC experiments for ggNesfatin-3 and hsNesfatin-3

Protein	Agent	c (mg/ml)	rmsd	*s*_20,w_	f/f_0_	MM_app_ (kDa)	Oligomerization state
**ggNesfatin-3**	EDTA (5 mM)	0.5	0.004678	1.53 2.17	2.12	26.1 43.8	Monomer (25%) Dimer (75%)
		0.75	0.005386	1.49 2.18	2.18	26.1 46.0	Monomer (24%) Dimer (76%)
		1.0	0.005703	1.53 2.19	2.26	28.5 49.0	Monomer (23%) Dimer (77%)
	Mg^2+^ (10 mM)	0.5	0.005294	2.28	1.89	39.7	Dimer (100%)
		0.75	0.005872	2.00 2.40	1.92	33.4 43.9	Monomer (17%) Dimer (83%)
		1.0	0.006543	1.88 2.40	1.93	30.9 44.5	Monomer (8%) Dimer (92%)
	Ca^2+^ (10 mM)	0.5	0.004854	1.94 2.44	1.72	27.0 38.2	Monomer (26%) Dimer (74%)
		0.75	0.005517	1.91 2.50	1.80	28.2 42.4	Monomer (24%) Dimer (76%)
		1.0	0.006183	1.89 2.53	1.81	28.1 43.4	Monomer (23%) Dimer (77%)
	Zn^2+^ (100 µM)	0.5	0.005252	1.95 2.48	1.70	26.9 38.7	Monomer (26%) Dimer (74%)
		0.75	0.006271	1.82 2.45	1.78	26.0 41.0	Monomer (20%) Dimer (80%)
		1.0	0.005493	1.70 2.41	1.85	24.8 42.0	Monomer (21%) Dimer (79%)
	Zn^2+^ (300 µM)	0.5	0.005293	2.54 3.08	1.58	35.5 47.4	Monomer (41%) Dimer (59%)
		0.75	0.005616	2.50 3.06	1.59	35.4 47.9	Monomer (36%) Dimer (64%)
		1.0	0.006104	2.37 2.91	1.62	33.6 45.6	Monomer (30%) Dimer (70%)
	Zn^2+^ (800 µM)	0.5	0.005559	2.46 3.49	1.52	32.1 54.2	Monomer (10%) Dimer (90%)
		0.75	0.006844	2.46 3.49	1.52	32.0 54.3	Monomer (9%) Dimer (91%)
		1.0	0.006807	2.50 3.35	1.52	33.2 53.7	Monomer (12%) Dimer (88%)
**hsNesfatin-3**	EDTA (5 mM)	0.5	0.005253	2.44	2.19	56.1	Dimer (100%)
		0.75	0.005695	2.42	2.28	58.6	Dimer (100%)
		1.0	0.006596	2.41	2.38	62.2	Dimer (100%)
	Mg^2+^ (10 mM)	0.5	0.005388	2.55	2.16	59.0	Dimer (100%)
		0.75	0.005716	2.55	2.16	58.6	Dimer (100%)
		1.0	0.006644	2.55	2.23	61.7	Dimer (100%)
	Ca^2+^ (10 mM)	0.5	0.005404	2.32 2.80	1.84	40.1 53.4	Monomer (13%) Dimer (87%)
		0.75	0.005823	2.25 2.80	1.89	39.8 55.1	Monomer (9%) Dimer (91%)
		1.0	0.008080	2.07 2.76	1.93	36.3 55.6	Monomer (4%) Dimer (96%)
	Zn^2+^ (100 µM)	0.5	0.007259	3.14	1.47	45.1	Dimer (100%)
		0.75	0.008686	3.19	1.54	49.4	Dimer (100%)
		1.0	0.009081	3.03 4.93	1.64	50.1 104.0	Dimer (98%) Tetramer (2%)
	Zn^2+^ (300 µM)	0.5	0.007857	3.65	1.38	51.4	Dimer (100%)
		0.75	0.007742	2.90 3.61	1.41	37.7 52.2	Monomer (11%) Dimer (89%)
		1.0	0.010962	3.50	1.41	49.6	Dimer (100%)
	Zn^2+^ (800 µM)	0.5	0.007036	3.94	1.31	53.6	Dimer (100%)
		0.75	0.007492	3.93 5.78	1.36	55.8 99.6	Dimer (99%) Tetramer (1%)
		1.0	0.008561	3.92 5.73	1.36	55.0 97.0	Dimer (99%) Tetramer (1%)

Numbers in brackets indicate the percentage of each fraction and were determined by considering 100% for the sum of the main indicated types of sedimenting species. rmsd – root-mean-square deviation; *s*_20,w_ – sedimentation coefficient under standard conditions (i.e., water, 20 °C); f/f_0_ – frictional ratio (the ratio of the actual frictional coefficient to that for an anhydrous sphere with an equal volume); MM_app_ – apparent molecular weight derived from SV-AUC experiments

In contrast, apo-hsNesfatin-3 exists in solution mostly in one oligomeric state. In this case, the value of the sedimentation coefficients (Table 2) of the only and very well-defined peak corresponded to the mass of the dimer (MM_app_ of 56–62 kDa). Further analysis revealed that neither the shape nor the oligomerization state of holo-hsNesfatin-3 was changed in the presence of Mg^2+^ (Fig. 6F). In the presence of Ca^2+^, two significant species were detected, with the principal component (at 2.76–2.80 S) being again consistent in size with that expected for the dimer (Table 2). The second population (2.07–2.32 S; 4–13%), composed of lower molecular weight species, seemed to reflect the presence of the monomeric state of hsNesfatin-3. Moreover, its lesser contribution decreased as the concentration of the protein increased, which indicated that Ca^2+^-hsNesfatin-3 exists in solution as a dimer in rapid equilibrium with smaller oligomeric forms. The most pronounced differences between the sedimentation profiles of apo- and holo-states of hsNesfatin-3 were observed after the addition of Zn^2+^ (Fig. 6H–J), after which the observed peaks appeared to be less prominent and considerably broader than those of the apo-protein samples. Although solutions of Zn^2+^-hsNesfatin-3 were dominated by species with a molecular mass consistent with that of a dimer (Table 2), trace amounts of higher molecular weight oligomers were also detected at 5.73–7.78 S (the putative tetramer; 1%) but only for an 800-μM solution of ZnCl_2_. Interestingly, as the concentration of Zn^2+^ was increased, the distribution peak gradually shifted toward higher *s* values (Fig. 6C–J), especially compared to those obtained for apo-hsNesfatin-3 (Table 2).

The SV-AUC analysis was able to determine not only the sedimentation coefficients of the sedimenting species but also the frictional ratios, f/f_0_ (i.e., the frictional coefficients of the proteins compared to that of an ideal sphere of identical volume). According to the collected data, the shape of both apo-ggNesfatin-3 and apo-hsNesfatin-3 molecules deviated from a sphere and adopted rather extended conformations (f/f_0_ of 2.12 and 2.07 for 0.5 mg/ml gg-Nesfatin-3 and hs-Nesfatin-3, respectively). Moreover, the obtained results showed a concentration-dependent increase in the f/f_0_ values (up to 2.26 and 2.38 for 1.0 mg/ml proteins, respectively). In the presence of different divalent metal ions, the f/f_0_ values obtained for ggNesfatin-3 consequently decreased from 1.89–1.93 (Mg^2+^), through 1.72–1.81 (Ca^2+^) to 1.52 (800 μM Zn^2+^), indicating a conformational change to a more compact or more globular-like structure, which correlated well with the limited proteolysis and fluorescence data discussed above. The conformation of hsNesfatin-3 appeared to be less susceptible to the presence of Mg^2+^ (f/f_0_ of 2.16–2.23). However, the tendency for compaction was retained in both Ca^2+^-hsNesfatin-3 (f/f_0_ of 1.84–1.93) and especially in Zn^2+^-hsNesfatin-3 (f/f_0_ of 1.31–1.36).

In summary, based on the hydrodynamic properties determined by SV-AUC, we can conclude that both nesfatins exist in solution as extended molecules composed of both, structured and intrinsically disordered regions and with the propensity for substantial conformational changes upon metal ion binding.

### Thermodynamics of the interactions of the nesfatin-3s with divalent metal ions

**Fig. 7:**
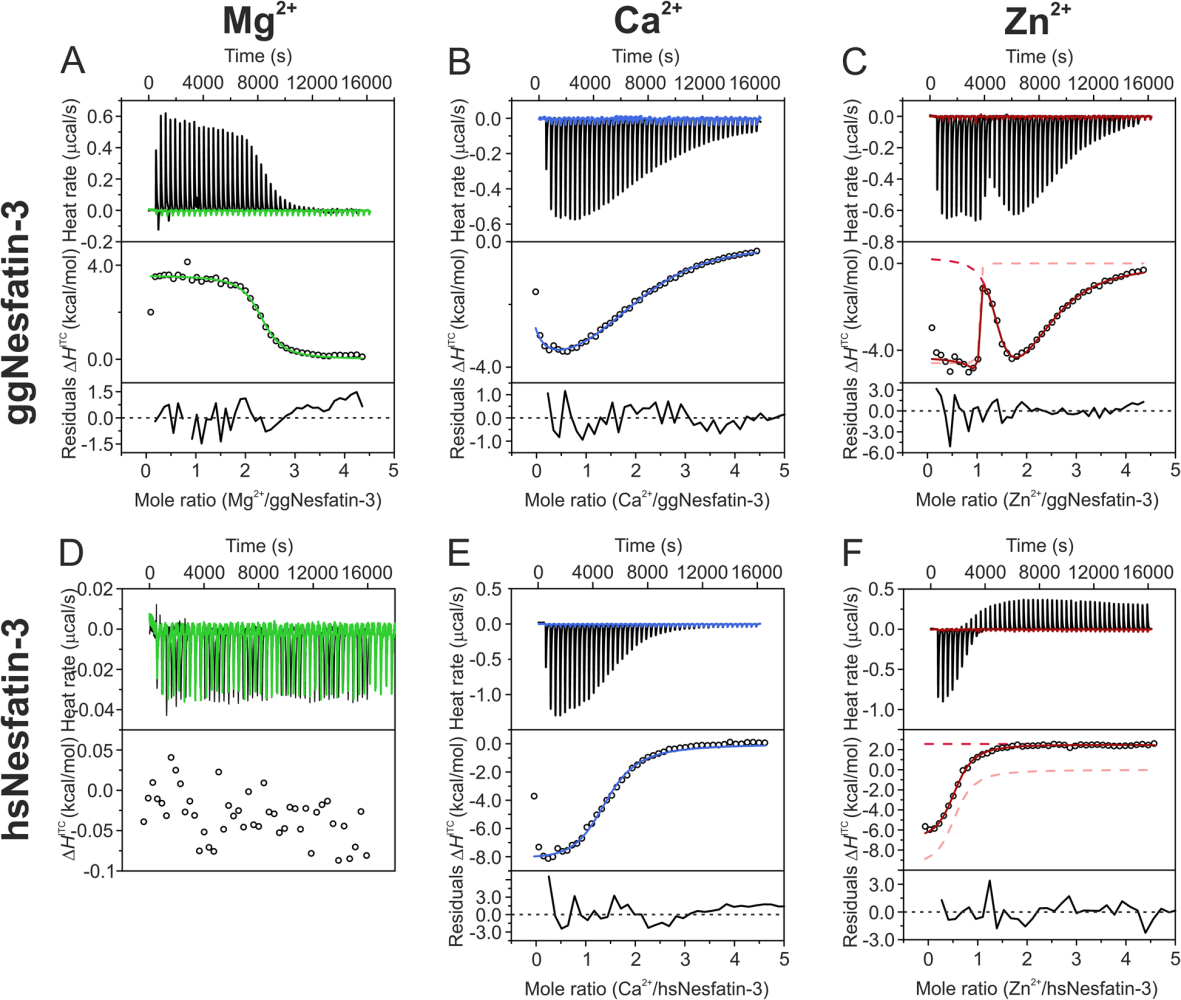
ITC analysis of Mg^2+^ (**A**, **D**), Ca^2+^ (**B**, **E**) and Zn^2+^ (**C**, **F**) binding to ggNesfatin-3 (**A**–**C**) and hsNesfatin-3 (**D**–**F**). The top panels present the thermograms corrected for the baseline, and the color lines show the control titrations. The middle panels represent the binding isotherm with the fitted models. Residuals are shown at the bottom of each panel. For the titration of Zn^2+^ to ggNesfatin-3 (**C**), the sum of the single site (light red dashed line) and sequential two site (red dashed line) models is presented as a dark red solid line. In the case of Zn^2+^ titration on hsNesfatin-3 (**F**), the sum of the single site (light red dashed line) and linear blank (red dashed line) models is presented as a dark red solid line. All experiments were performed in 20 mM Hepes (pH 7.5), 150 mM NaCl at 25 °C

ITC measurements were performed to shed light on the thermodynamic parameters of Mg^2+^, Ca^2+^ and Zn^2+^ ion interactions with nesfatin-3s. Mg^2+^ titration on ggNesfatin-3 showed a simple, monophasic, binding isotherm with the transition at a ratio ~ 2, suggesting that ggNesfatin-3 binds two Mg^2+^ ions per molecule with quite a high affinity (*K*_d_ of 2.4 μM) (Fig. 7A). The Mg^2+^ process appeared to be enthalpically unfavorable (Δ*H* of 3.47 kcal/mol) and entropically driven. The evaluated entropic factor was noticeably high (TΔ*S* of 11.24 kcal/mol) to compensate for the endothermic effect accompanying Mg^2+^ binding (Table 3). The large entropic effect may suggest that Mg^2+^ binding influences the monomer–dimer equilibrium of ggNesfatin-3. The binding of Ca^2+^ to ggNesfatin-3 was represented by a thermogram showing two exothermic processes (Fig. 7B). The obtained result was fitted to a sequential model and showed that ggNesfatin-3 binds two Ca^2+^ ions per monomer (Table 3). The first binding event occurred with a dissociation constant of 116 μM and a binding enthalpy of -6 kcal/mol. The second occurred with a much higher affinity, characterized by a dissociation constant of 45.9 μM and a similar enthalpy change of -6.36 kcal/mol. The corresponding entropic factors of both binding sites were found to be lower values, which suggested no significant oligomeric state changes induced by Ca^2+^ binding. The Zn^2+^ interaction with ggNesfatin-3 was more complex and characterized by a more convoluted binding isotherm, showing multiple processes with high affinities and diverse enthalpy changes (Fig. 7C and Table 3). The data collected for Zn^2+^-saturated ggNesfatin-3 were fitted simultaneously with both an independent (one-site) model and a multiple independent site model (which assumes that molecules have multiple noncooperative binding sites). Here, the first binding event was found to be exothermic (Δ*H*_1_ of -4.57 kcal/mol) and characterized by a constant at least lower than 7.2 nM; however, the exact stability constant was uncertain, as ITC is not a suitable method for measuring such high-affinity interactions. This binding event was additionally accompanied by structural changes causing large entropic gain, with an entropic factor TΔ*S*_1_ of 6.54 kcal/mol. The second binding event was characterized by a higher dissociation constant of 0.42 μM, a small endothermic effect, and an even larger gain in entropy value (TΔ*S*_2_ of 8.97 kcal/mol), suggesting that the reaction was driven by some hydrophobic effect possibly caused by a structural rearrangement or formation of oligomeric complexes. The third event was found to be enthalpically driven and was entropically not favorable. Although the combination of both independent and multiple independent models did not assume that the events appeared in sequential order, substantial differences between *K*_d_ values (above three orders of magnitude) allow us to assume that the first binding event may occur before the two subsequent events and that the utilized model explains well the observed binding isotherm.

**Table 3:** Thermodynamic parameters of Mg^2+^, Ca^2+^, and Zn^2+^ ion complexation with nesfatin-3s derived from ITC data

	**ggNesfatin-3**			**hsNesfatin-3**	
	**Mg**^**2+**^	**Ca**^**2+**^	**Zn**^**2+**^	**Ca**^**2+**^	**Zn**^**2+**^
Model	Single site	Sequential two site	Single site and two independent sites	Single site	Single site, linear blank
[nesfatin-3] (µM)	120	95	120	85	80
[M^2+^] (µM)	2000	2000	2000	2000	2000
n_1_	2.30 ± 0.02	–	1.01 ± 0.01	2.08 ± 0.04	0.99 ± 0.02
*K*_d1_ (µM)	2.4 ± 0.4	116 ± 81	7 × 10^–3^ ± 24 × 10^–3^	9 ± 1	7 ± 1
Δ*H*_1_ (kcal/mol)	3.47 ± 0.05	-6 ± 2	-4.57	-8.3 ± 0.2	-9.8 ± 0.4
TΔ*S*_1_ (cal/mol)	11,241	-187	6541	-1374	-2736
Intercept (µcal)	–	–	–	–	26 ± 2
Slope	–	–	–	–	-0.14 ± 0.05
n_2_	–	–	1.35 ± 0.06	–	–
*K*_d2_ (µM)	–	45.9 ± 16.1	0.42 ± 0.29	–	–
Δ*H*_2_ (kcal/mol)	–	-6.36 ± 2.06	0.28 ± 0.87	–	–
TΔ*S*_2_ (cal/mol)	–	-437	8968.35	–	–
n_3_	–	–	1.12 ± 0.19	–	–
*K*_d3_	–	–	31.11 ± 9.17	–	–
Δ*H*_3_ (kcal/mol)	–	–	-7.414 ± 1.83	–	–
TΔ*S*_3_ (cal/mol)	–	–	-1266	–	–

M^2+^ – metal ion (Mg^2+^, Ca^2+^ or Zn^2+^), *K*_d1/d2/d3_ – dissociation constant for the first, second or third binding site, Δ*H*_1/2/3_ – enthalpy of complex formation for the first, second or third binding site, TΔ*S*_1/2/3_ – entropic factor for the first, second or third binding site

ITC was also attempted for hsNesfatin-3. The data collected for Mg^2+^-titrated hsNesfatin-3 revealed that the heat changes were too small, even at the highest concentrations of the protein and metal ions used (Fig. 7D), and therefore it was not pursued. The binding of Ca^2+^ to hsNesfatin-3 (Fig. 7E) was found to be different from the binding of Ca^2+^ to ggNesfatin-3 (Fig. 7B). The fitting showed that the binding of two Ca^2+^ ions to hsNesfatin-3 is mostly enthalpy-driven (Δ*H* of -8.3 kcal/mol) and is characterized by a slightly lower affinity (*K*_d_ of 9 μM) than Zn^2+^ (Table 3). In contrast to ggNesfatin-3, hsNesfatin-3 also showed different behavior in the case of Zn^2+^ binding. Zn^2+^ interaction with the human homolog was initially observed as substantial heat release with an inflection point at a Zn^2+^:hsNesfatin-3 ratio of approximately 1, above which an unfavorable endothermic signal was observed even in the presence of a high excess of Zn^2+^ (Fig. 7F and Table 3). Because the titration curve showed that the protein binding sites might not be completely saturated with Zn^2+^, the points near the end of the titration were linearly fitted and subtracted from the raw data. Therefore, although we consider the values for the stoichiometry of binding (n) and the micromolar affinity (*K*_d_ of 7 μM) reasonable, nevertheless, either the enthalpy or the calculated entropy values should be treated with caution.

## Discussion

As an irreversible modification, proteolytic processing of full-length proteins still represents an underappreciated, albeit essential, posttranslational modification (PTM), raising many unanswered questions about the significance of the structure of the precursor proteins, especially regarding the anticipated three-dimensional structure (and resulting function) of the isolated peptides that are derived from it. The hydrolysis of precursor proteins often yields peptides with different, even antagonistic, activities, highlighting the relevance of PTM events [31]. As a consequence, an increasing number of such peptides are, in turn, recognized as important peptide hormones that influence or coordinate distinct cellular functions. The data show that many potential biomarkers of diseases are stable proteolytic fragments in biological fluids, including such examples as the C-terminal fragment of albumin (Pro1708/Pro2044) [32], the ectodomain of human epithelial growth factor receptor-2 (HER2 rb2) [33], and a soluble fragment of cytokeratin 19 (CYFRA 21–1) [34]. Investigations, particularly of the structure of the secreted proteins, are consequently important to define their new functions or potential regulation sites as highly promising drug targets for the future.

Although knowledge of Nucb2/nesfatin-1 has greatly increased in recent years, the biological roles, if any, of nesfatin-3 and the role of divalent metal ions that would be able to bind to it remain unknown. Our comprehensive structural analysis of the nesfatin-3s revealed several important findings. The first is the susceptibility of their secondary structures to metal ion-induced conformational changes. This was particularly clearly observed as a pronounced increase in the α-helical fraction of Ca^2+^-bound hsNesfatin-3 (Table 4). We did not observe such a tendency for hsNucb2 [16], which indicates that the isolated C-terminal part of hsNucb2 in solution adopts a different conformation than that of the full-length precursor protein. Ca^2+^ was also the main ion affecting the secondary structure of ggNesfatin-3, albeit not to such an extent. Puzzled by this discrepancy, we wondered whether the differences within the tertiary structures of the nesfatin-3s would also be observed, highlighting the importance of both seeking and understanding the effect of the processing of Nucb2s. The results of the limited proteolysis showed that both Ca^2+^ and Zn^2+^ impacted the susceptibility to the protein digestion of both nesfatin-3s but in different ways. In the presence of a high concentration of Ca^2+^ and Zn^2+^, the rate of hydrolysis of both proteins decreased significantly, making hsNesfatin-3 especially resistant to proteolysis. Additionally, the lowest concentration of Ca^2+^, which induced the conformational changes of nesfatin-3s, was different for both homologs. For hsNesfatin-3, the critical concentration of Ca^2+^ (0.1 mM) was tenfold smaller than that for ggNucb2 (1.0 mM), indicating differences in the binding affinity. Notably, the presence of a high concentration of Zn^2+^ was found to be problematic for both proteins. It reduced the stability of ggNesfatin-3, presumably leading to its precipitation. The proteolysis pattern, regardless of the metal ion used, revealed the additional generation of similar populations of peptides. One possible explanation for these results is that most of the amino acid residues undergoing cleavage within the nesfatin-3 sequence might be located at the C-terminal IDR. In contrast, during our previously reported limited proteolysis analysis [16, 17], we observed dramatic changes in the digestion patterns for both full-length nucleobindins saturated with either Ca^2+^ or Zn^2+^. Of note, the impact of Mg^2+^ was observed but mostly on the structure of ggNesfatin-3. We characterized the process of Mg^2+^ binding to ggNesfatin-3 as entropically driven. Such a huge entropic effect might suggest that Mg^2+^ binding influenced the oligomeric state of ggNesfatin-3. On the other hand, the homologous hsNesfatin-3 titrated with Mg^2+^ did not show any enthalpy changes that could suggest the interaction of hsNesfatin-3 with Mg^2+^. In fact, we observed almost no response of hsNesfatin-3 after Mg^2+^ addition, whereas Mg^2+^-binding to ggNesfatin-3 triggered a global conformational change in the tertiary/quaternary (rather than secondary) structure, which was also reflected in a quite low *K*_d_ value (Table 4). Such behavior might be a consequence of subtle structural differences. ggNesfatin-3 possesses an additional 30 amino acid residues at the C-terminus, which probably impacts the whole structure and molecular properties of the protein. Moreover, the differences within sequences of EF-hand domains seem to exert a significant switch of their ligand binding affinities. While there is an Arg residue, instead of Gly, at position 6 of the second EF-hand motif of hsNesfatin-3 (making the sequence of the domain rather noncanonical), in the ggNesfatin-3 sequence, there are two noncanonical EF-hand motifs, with Arg residues at position 6. This suggests that a single amino acid substitution within the divalent metal ion binding domain may subtly alter the structure of the region, enabling different ligands to take up coordinating positions [35]. Free Ca^2+^ ion concentrations vary constantly inside and outside the cell. In contrast, the free Mg^2+^ ion concentration in cells is high and practically constant [36]. Mg^2+^ competes for most Ca^2+^ binding sites. For this reason, Ca^2+^ binding sites are conventionally subdivided into sites binding both Ca^2+^ and Mg^2+^ and sites specific only for Ca^2+^, based on the values of the dissociation constants [37]. Eventually, to evaluate Ca^2+^/Mg^2+^ binding propensity, we applied the AlphaFill approach (not shown). The algorithm uses sequence and structure similarity to ‘transplant’ small molecules and ions from experimentally determined structures to predicted protein models [38]. Remarkably, the predictor transplanted two Mg^2+^ and two Ca^2+^ (originating from the PDB structures 5VKW and 1TCF, respectively) to the AlphaFill models of ggNesfatin-3 and hsNesfatin-3, respectively, correctly highlighted the differences between analyzed nesfatins. The affinity for Mg^2+^ not only distinguishes the nesfatin-3s from each other but also distinguishes them from full-length nucleobindins, implying their different physiological roles. Perhaps Mg^2+^ binding with high affinity requires both the favorable modification of two EF-hand domain sequences and some specific surface features that are accessed only after proteolytic conversion of the full-length Nucb2s.

**Table 4:** Significant differences in the structures of nesfatin-3s in relation to the structures of Nucb2s

Analyzed feature	ggNesfatin-3	hsNesfatin-3	ggNucb2	hsNucb2
α-helix-to-random coil ratio for apo-protein	1	1.2	1.7	2.8
α-helix-to-random coil ratio in the presence of:				
Mg^2+^	1.3	1.5	1.6	3.1
Ca^2+^	1.7	2.6	2.2	2.7
Zn^2+^	1	1.6	0.4	3.7
Sensitivity of the apo-protein to the proteolytic hydrolysis	moderate	low	high	moderate
Sensitivity of the holo-protein (compared to the apo-protein) to the proteolytic hydrolysis in the presence of:				
Mg^2+^	↓	→	→	↓
Ca^2+^	↓	↓	↓	↓
Zn^2+^	↓	↓	↓	↓
Dimer-to-monomer ratio for the apo-protein at the highest used protein concentration	3:1	dimer only	monomer only	dimer only
Dimer-to-monomer ratio for the holo-protein at the highest used protein concentration and in the presence of:				
Mg^2+^	9:1	dimer only	1:12	9:1
Ca^2+^	3:1	24:1	1:3	dimer only
Zn^2+^	3:1–9:1	dimer only	mix	6:1
The *K*_d_ values estimated after the binding of:				
Mg^2+^	2.4 µM	n.d	230 µM	203.2 µM
Ca^2+^	116.3 µM/45.9 µM	8.79 µM	190 µM/73 µM	16.8 µM/19.8 µM
Zn^2+^	7.21 nM/0.4 µM/31.1 µM	6.95 µM	188 µM/34.4 µM	23.9 µM/14.3 µM

The data presented for ggNucb2 and hsNucb2 were adopted from [16, 17]. Arrows indicate the stimulatory (↑), suppressive (↓), or neglected ( →) effects listed in the left column; mix – many different oligomeric forms; n.d. – not determined

We then used SV-AUC analysis to determine how divalent metal ions may contribute to the overall structure of nesfatin-3s. The hypothesis that nesfatin-3s may dimerize resulted naturally from the fact that the EF-hand domains, located centrally within their structures, tend to dimerize for functionality in solution [39]. This is, however, not a rule. Indeed, hsNesfatin-3 exclusively formed dimers, and only for a Ca^2+^-bound protein were a small fraction of monomers observed. Structural analysis of ggNesfatin-3 was found to be more complex but also revealed that the protein exists in solution mostly as a dimer, and the fraction of the dimeric forms increased significantly for Mg^2+^-saturated ggNesfatin-3. Alternatively, apo-ggNucb2 behaved as a monomer in the solution [16, 17], whereas ggNucb2 in the presence of Mg^2+^ consisted mainly of monomeric and a low amount of dimeric species ([17] and Table 4). This suggests that the dimerization of ggNesfatin-3 is feasible only in the absence of nestatin-1/2 and might be supported by contacts of the isolated carboxyl-terminal-half region of ggNucb2 molecules. The SV-AUC data, together with the fluorescence and limited proteolysis analyses, provided a general sense of how the rod-like α-helical/disordered nesfatin-3s change conformations upon metal ion binding. We noticed that in solution, both homologs adopted much more closed conformations in their holo-forms, especially as a result of Zn^2+^ binding.

The two nesfatin-3s analyzed here, regardless of the organism that was the source of the protein sequence, appear to have the same basic core structure common to both proteins. Our CD data demonstrated that they are one-third α-helical and one-third intrinsically disordered and interact with the same set of metal ions. In their apo forms, they showed similar shapes in SV analysis and existed in solution as extended molecules with the propensity for compaction in the presence of divalent metal ions, as discussed above. Nevertheless, the differences among nesfatin-3s are informative. Each favored interaction with a different metal ion and displayed unique binding affinities, as demonstrated with ITC. ggNesfatin-3 showed a unique affinity toward Zn^2+^ and preferred Mg^2+^ binding over Ca^2+^, whereas hsNesfatin-3 bound Ca^2+^ in a model way. It is intriguing that although Mg^2+^ binding was observable for hsNucb2, it was barely observed for hsNesfatin-3. Our data also demonstrated the pronounced effect of Zn^2+^-binding on the structure of both nesfatin-3s: we observed precipitation of ggNesfatin-3 and oligomerization/aggregation of hsNesfatin-3 at high Zn^2+^ concentrations. The hsNesfatin-3-Zn^2+^ interaction also seemed to be accompanied by some additional process, which needs to be identified. Perhaps this will help us better understand the main physiological function of nesfatin-3 in the future. Our structural analysis will continue to yield insight into the mechanism of action of this exciting protein. The story of nesfatin-3 has just begun.

## Data Availability

The data that support the findings of this study are available on request from the corresponding author. The data are not publicly available due to privacy or ethical restrictions.

## Abbreviations

CD: Circular dichroism spectroscopy
IDPs: Intrinsically disordered proteins
IDRs: Intrinsically disordered regions
ITC: Isothermal titration calorimetry
MM: Molecular mass
Nucb2: Nucleobindin-2
PC1/3: Prohormone convertase 1/3
PC2: Prohormone convertase 2
PTM: Posttranslational modification
SV-AUC: Sedimentation velocity analytical ultracentrifugation
ZIP: The leucine zipper motif
